# Genome Sequence of Fowl Aviadenovirus A Strain JM1/1, Which Caused Gizzard Erosions in Japan

**DOI:** 10.1128/genomeA.00749-17

**Published:** 2017-10-12

**Authors:** Khompakorn Thanasut, Kan Fujino, Motoko Taharaguchi, Satoshi Taharaguchi, Fumie Shimokawa, Masaru Murakami, Kozo Takase

**Affiliations:** a Laboratory of Veterinary Microbiology II, Department of Veterinary Medicine, School of Veterinary Medicine, Azabu University, Fuchinobe, Chuo-ku, Sagamihara-shi, Kanagawa, Japan; b Division of Experimental Animal Research, National Institute of Infectious Diseases, Shinjuku-ku, Tokyo, Japan; c Laboratory of Molecular Biology, Department of Veterinary Medicine, School of Veterinary Medicine, Azabu University, Fuchinobe, Chuo-ku, Sagamihara-shi, Kanagawa, Japan; d Laboratory of Veterinary Microbiology, Department of Veterinary Medicine, Joint Faculty of Veterinary Medicine, Kagoshima University, Kagoshima, Japan

## Abstract

This study reports the complete genome sequence of fowl aviadenovirus A strain JM1/1, which caused gizzard erosions in broilers occurring in Japan. The JM1/1 genome is 43,809 bp in length and most closely related to the strain chicken embryo lethal orphan (CELO); moreover, multiple site insertions and deletions were found.

## GENOME ANNOUNCEMENT

Fowl aviadenovirus is ubiquitous in poultry and is a leading cause of worldwide economic loss; it has been isolated from both sick and healthy chickens. In chickens, fowl aviadenovirus can cause inclusion body hepatitis, hydropericardium syndrome, egg drop syndrome, and respiratory tract disease. Chickens are infected by both vertical and horizontal transmission via secretions or excretions. An outbreak of a fowl aviadenovirus strain causing gizzard lesions was the first natural outbreak in layer chickens in Japan in 1993 (1). Subsequently, epidemics occurred more recently in East Asia (especially Japan and South Korea) and Europe (2–8). In Japan, isolates obtained from poultry showing gizzard erosions belong to the family *Adenoviridae* and genus *Aviadenovirus*.

Seeding viral stock obtained from broiler chickens with gizzard erosions was detected at a slaughterhouse in Kagoshima, Japan, in 2000. Viruses were isolated using chicken embryonic liver cells. Isolates were identified as serotypes 1 and 8 using a neutralization method; only the JM1/1 isolate strain of serotype 1 induced gizzard erosions in an experimental infection with specific-pathogen-free (SPF) chickens (9). In this study, the complete genome sequence of fowl aviadenovirus A strain JM1/1 was determined.

Viruses were propagated on a monolayer of chicken kidney cells prepared from 3- to 14-day-old SPF chicks, cultured in Eagle minimal essential medium (EMEM [Nissui Pharmaceutical Co., Tokyo, Japan]) containing 5% fetal bovine serum (MP Biomedicals, Solon, OH), incubated at 37°C under a 5% CO_2_ atmosphere, and subjected to DNA extraction using the phenol-chloroform DNA extraction method. For complete-genome sequencing, next-generation sequencing was used. DNA was fragmented to create smaller strands and then ligated with adaptors and amplified using emulsion PCR. Sequencing was performed using Ion Torrent, the hydrogen ions produced during DNA polymerization were detected, and the whole-genome sequences were created. The sequences were aligned and edited using Integrative Genetic Viewer (IGV) version 2.3.79 and the Genetyx program.

The complete genome of JM1/1 was found to be 43,809 nucleotides long, with 54.31% G+C content. Compared to other sequences available in GenBank, JM1/1 revealed 99% nucleotide sequence identity to Avian adenovirus chicken embryo lethal orphan (CELO) (GenBank accession number U46933). Interestingly, the nucleotide difference compared to CELO showed multiple site insertions and deletions, including in open reading frames (ORFs) 10, 11, 14, 18, 19, and 21 and the L4 and L5 genes, indicating that these positions may serve as nonfunctional genes and ORFs due to changes in protein translation. Moreover, the tandem repeat sequence was assessed by the Tandem Repeats Finder program, which showed one repeat with a 12-base CCCCCTGTACAC consensus sequence, compared to CELO, in which 2 repeats were found.

In summary, the complete genome sequence of fowl aviadenovirus A strain JM1/1 was determined, and it was found that JM1/1 is a member of serotype 1 and is nearly identical to the strain CELO, which is a fowl aviadenovirus serotype 1 European strain; furthermore, multiple site insertion and deletion differences were found, and these will provide more information on the evolution of fowl aviadenovirus and may help elucidate viral pathogenesis on molecular biology.

### Accession number(s).

The nucleotide sequence of fowl aviadenovirus A strain JM1/1 has been submitted to GenBank under the accession number MF168407.
